# Wind energy-driven rabbit hair TENG for railway detection

**DOI:** 10.1016/j.isci.2026.116335

**Published:** 2026-06-11

**Authors:** Linchao Chen, Lin Zhang, Shipeng Wang, Baoping Wang, Yubin Qi, Da Zhao

**Affiliations:** 1 School of Construction Machinery, Shandong Jiaotong University, Jinan 250357, China; 2 Shandong Key Laboratory of Technologies and Systems for Intelligent Construction Equipment, Shandong Jiaotong University, Jinan 250357, China; 3 Shandong Provincial Engineering Research Center for Transportation Construction Equipment Technology and Intelligent Construction, Shandong Jiaotong University, Jinan 250357, China

**Keywords:** energy engineering, energy sustainability, materials science

## Abstract

To improve the output performance and durability of triboelectric nanogenerators (TENGs), this study presents a high-performance flexible-contact TENG (FC-TENG). The FC-TENG consists of an energy-harvesting unit and a power-generation unit. Furthermore, the performance of the power-generation unit is analyzed using the nodal analysis method and validated through simulation. Notably, through experimental validation, the structural parameters of the FC-TENG are optimized, and the output performance under parallel connection is systematically investigated. The results indicate that, at a rotational speed of 300 rpm, the open-circuit voltage, short-circuit current, transferred charge, and output power reach 2,284 V, 45.16 μA, 798.38 nC, and 10.68 mW, respectively. After 3.45 million operating cycles, the transferred charge of the power-generation unit decreases from 221.58 to 185.61 nC, corresponding to a degradation of only 7%. Overall, this study achieves a balance between output performance and durability, offering an efficient solution for railway line monitoring applications.

## Highlights

• A high-performance flexible-contact TENG (FC-TENG) based on rabbit hair is proposed
• Through optimizing the structure, the voltage of the FC-TENG reaches 2,284 V
• A single power-generation unit exhibits long-term durability of 3.45 million cycles

## Graphical abstract

**Figure:**
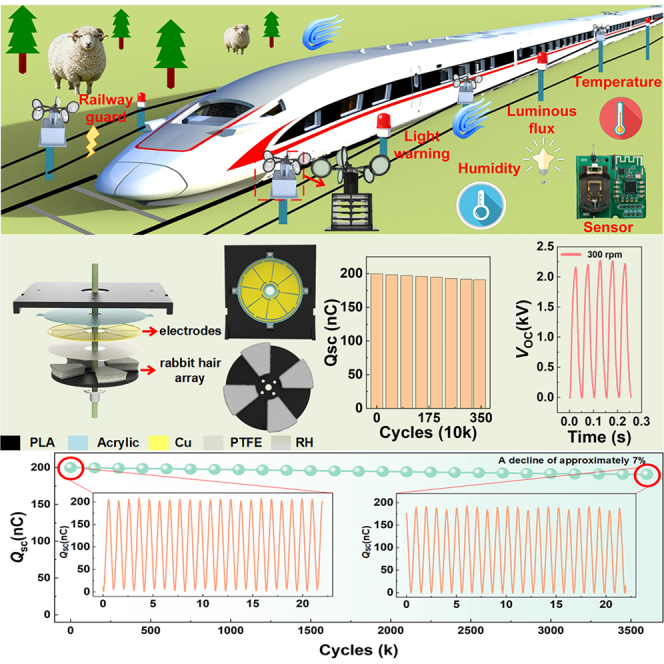

## Introduction

With the rapid development of high-speed railway systems, operational safety has become a critical concern.^1^^,^^2^ Railway lines extend across vast geographic regions and are exposed to harsh environmental conditions, including strong winds and extreme temperature fluctuations, thereby necessitating continuous real-time monitoring. Moreover, a significant portion of railway networks passes through remote and uninhabited areas, where wildlife intrusion onto tracks presents a serious risk to infrastructure safety. Although continuous monitoring and active protection systems are essential, power supply remains a major bottleneck.^3^ Given that railway networks span hundreds to thousands of kilometers, there is an urgent demand for low-cost, distributed, self-powered sensing solutions. In addition, as essential infrastructure, railway lines and their supporting facilities must exhibit high durability and long-term operational stability.

Since the triboelectric nanogenerator (TENG) was first proposed in 2012, its distinctive energy conversion mechanism based on Maxwell’s displacement current has demonstrated substantial potential for harvesting low-frequency mechanical energy, including wind energy, vibrational energy, and human motion energy.^4^ Compared with traditional electromagnetic generators, TENGs offer several advantages, such as high output voltage, flexible structural design, and low manufacturing cost. As a result, they are considered a promising candidate for self-powered systems in distributed outdoor applications.^5^ In recent years, significant progress has been made in optimizing TENG performance for specific application scenarios. For example, Han et al. developed a rabbit-hair (RH)-based flexible-contact TENG (FC-TENG) for smart agricultural monitoring.^6^ Li et al. developed an Ecoflex/poly(3,4-ethylenedioxythiophene)-poly(styrenesulfonate)(PEDOT:PSS)-based triboelectric nanogenerator (EP-TENG) with contact/non-contact bifunctional sensing for human-machine interaction. The device delivered a non-contact sensing range of 80 mm, a contact open-circuit voltage (*V*_OC_) of 124 V, and a power density of 75.3 mW/m^2^ and remained stable after 1,500 cycles, confirming its reliability for intelligent interactive applications.^7^ Fang et al. proposed a triboelectric-electromagnetic hybrid generator based on rotating conical rollers for wind energy harvesting, successfully powering low-power sensors.^8^ Hu et al. developed a stacked TENG structure with exceptional volumetric charge density and stability, providing an important reference for optimizing structural design and durability in our work.^9^ These studies collectively confirm the feasibility of TENGs for mechanical energy harvesting and self-powered systems, laying a solid foundation for their application in railway line monitoring.

Currently, most rotary TENGs used for wind energy harvesting adopt a rigid-contact configuration. However, the rigid friction between contacting materials leads to severe mechanical wear, which significantly compromises long-term device reliability.^10^^,^^11^ In contrast, FC-TENGs for wind energy harvesting have introduced innovative electrode structures, among which RH has been proposed as a promising triboelectric material. These electrodes exhibit favorable properties, including good durability and relatively high output performance.^12^^,^^13^ Nevertheless, their output characteristics are highly sensitive to structural parameters, which may lead to reduced energy-harvesting efficiency under suboptimal configurations. Moreover, in untrimmed RH structures, fiber shedding can occur, resulting in charge neutralization within the electrodes. This not only degrades output performance but also reduces device durability.^14^ Therefore, during wind energy harvesting, a trade-off exists between output performance and durability in FC-TENGs. Accordingly, there is an urgent need to rationally optimize electrode design to enhance the overall performance of FC-TENG systems.

To enhance the output performance and durability of flexible power-generating devices for railway line applications, RH is first adopted as a flexible electrode material, followed by systematic optimization of its structural parameters. By varying RH length, electrode spacing, and the number of RH layers, a high-performance power-generation unit with improved long-term stability is developed. This strategy effectively addresses both the underutilization of the performance potential of RH-based flexible electrodes and their limited durability, enabling their application in a prototype designed for wind energy harvesting along railway lines. This design not only improves the output performance and durability of conventional RH flexible electrodes but also achieves enhanced energy harvesting through parallel connection of multiple electrode plates. Accordingly, this study proposes a high-performance FC-TENG for railway wind energy harvesting. The electrode configuration and overall device structure are carefully designed, and a theoretical analysis of the output performance and equivalent circuit model is conducted. In addition, the performance of multiple power-generation units connected in parallel is systematically investigated. The output characteristics of the FC-TENG are experimentally evaluated under different conditions, including varying rotational speeds and load resistances. Finally, practical application tests are performed to supply power for temperature and humidity sensors, light flux sensors, and active safety protection systems, thereby demonstrating the applicability of the FC-TENG for railway line monitoring.

## Results and discussion

### Structural design and working mechanism

**Figure 1:**
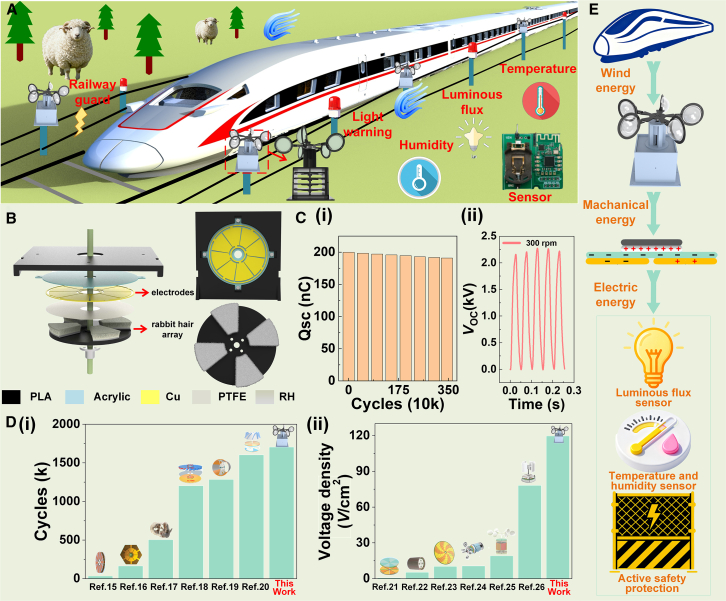
Working mechanism, structural design, and performance comparison of the FC-TENG (A) Application background. (B) Structure of a single power-generation unit. (C) Performance of the FC-TENG. (D) Performance comparison with other FC-TENGs. (E) Process of energy collection and storage application.

As shown in Figures 1A and S1, the FC-TENG can be applied for wind energy harvesting and safety protection along railway lines. The TENG units installed along the railway are capable of capturing wind energy, which serves as a power source for the system. This enables the operation of temperature-humidity sensors and light flux sensors, thereby supporting wireless data transmission. Meanwhile, a voltage of approximately 300 V is applied to the fence wire, effectively preventing humans and wild animals from crossing the barrier and thereby enhancing railway infrastructure safety. The FC-TENG consists of a polylactic acid (PLA) housing, four power-generation units, wind cups, and other structural components. As illustrated in Figure 1B, each power-generation unit comprises RH fixed onto an RH-supporting plate, a polytetrafluoroethylene (PTFE) film, and interdigitated copper electrodes mounted on an acrylic substrate. The use of RH enables flexible contact, which not only effectively reduces mechanical wear of friction materials but also enhances output performance by increasing the effective contact area. As shown in Figure 1C(i), when the FC-TENG operates at a rotational speed of 300 rpm, the *V*_OC_ reaches 2,284 V. Under the same operating condition, after 3.45 million cycles, the transferred charge (*Q*_SC_) of a single power-generation unit exhibits only a 7% degradation (Figure 1C[ii]). Figures 1D(i) and 1D(ii) present a comparison of durability^15^^,^^16^^,^^17^^,^^18^^,^^19^^,^^20^ and *V*_OC_^21^^,^^22^^,^^23^^,^^24^^,^^25^^,^^26^ with those of other rotary TENGs. Notably, the reported voltage values are normalized by electrode area (i.e., voltage divided by electrode area) to eliminate bias arising from variations in device size and electrode configuration. It is evident that, following parameter optimization, the FC-TENG demonstrates a superior voltage output. In addition to the normalized *V*_OC_, the FC-TENG also exhibits competitive performance in terms of short-circuit current (*I*_SC_) and *Q*_SC_ (Figures S3 and S4).

In contrast to previous studies, this work further optimizes key structural parameters, including RH length, inter-electrode spacing, and the number of RH patches. This optimization is intended to improve fiber length uniformity, suppress charge neutralization caused by hair shedding, and enhance both structural stability and charge transfer efficiency. As a result, an ultra-low performance degradation rate of only 7% after 3.45 million cycles is achieved. This performance surpasses that of rigid-contact TENGs and unoptimized RH-based TENGs, which typically exhibit a degradation rate exceeding 10% after 1.2 million cycles, while simultaneously increasing the output voltage to 2,284 V. Evidently, both the output voltage and long-term durability of the FC-TENG are significantly improved. Figure 1E presents a schematic illustration of the integration of the FC-TENG into a railway line monitoring system. By harvesting wind energy, the system is capable of supplying power to sensors as well as active safety protection devices.

### The theoretical analysis of the FC-TENG

**Figure 2:**
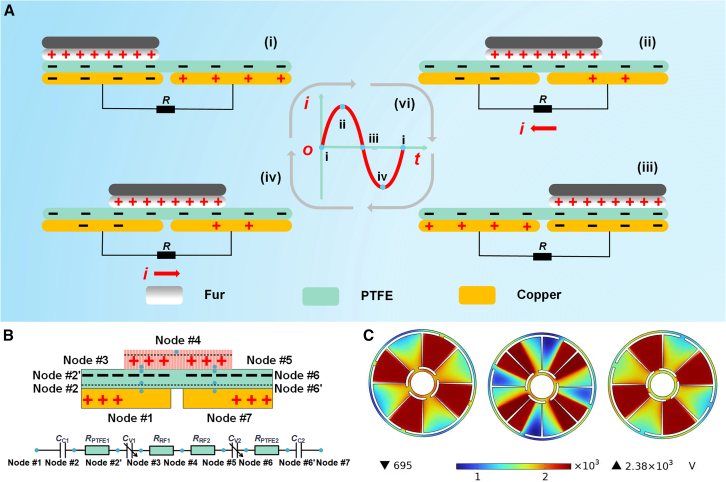
Charge transfer, node analysis, and simulation (A) Triboelectric charging, charge transfer process, and current-time curve of the FC-TENG. (B) Node analysis. (C) Finite element simulation of voltage distribution of interdigital electrodes.

The triboelectrification and charge transfer processes of the FC-TENG are illustrated in Figure 2A. These processes are described through four sequential states, which elucidate the temporal evolution of charge transfer and the underlying energy-harvesting mechanism.

A difference in electronegativity exists between RH and PTFE, with PTFE exhibiting higher electronegativity. Upon contact, triboelectrification occurs, whereby electrons are transferred from the RH to the PTFE surface. Consequently, PTFE becomes negatively charged, while the RH becomes positively charged. Simultaneously, positive charges are induced on the copper electrode through electrostatic induction (Figure 2A[i]). As the RH layer rotates to the intermediate position, the positive charges on the RH induce charge redistribution within the copper electrode. At this stage, a potential difference is generated in the external circuit due to the asymmetric charge distribution, driving directional electron flow (Figure 2A[ii]). When the RH and PTFE are nearly fully overlapped, the inductive effect of the positive charges on the copper electrode reaches its maximum, and the current gradually decreases to nearly zero, corresponding to the trough in the current profile (Figure 2A[iii]). During the separation of RH and PTFE, the electrostatic induction effect weakens. Accordingly, electrons previously accumulated on the copper electrode flow back to restore electrostatic equilibrium, producing a reverse current in the external circuit (Figure 2A[iv]). This process completes a full cycle when the two materials are fully separated and the charge distributions on all surfaces, including the copper electrode, return to their initial states (Figure 2A[i]). The current-time (*I*–*t*) profile (Figure 2A(vi]) clearly illustrates the alternating current behavior throughout the cycle.

Figures 2B and S2 present the equivalent circuit schematic of the FC-TENG constructed using the nodal analysis method.^27^^,^^28^ The internal electrical characteristics of the TENG are first analyzed using nodal theory, based on which an equivalent circuit model is established. On this foundation, a theoretical framework describing the output behavior of the TENG is developed. As shown in Figure 2B, nodes 1 and 2, as well as nodes 6′ and 7, form constant capacitors *C*_C1_ and *C*_C2_, respectively. Triboelectric interfaces are formed between nodes 2 and 2′, nodes 3 and 4, nodes 4 and 5, and nodes 6 and 6′, where surface charge transport and dissipation can be represented by internal resistances *R*_PTFE1_, *R*_RF1_, *R*_RF2_, and *R*_PTFE2_, respectively. In addition, two variable capacitors, *C*_V1_ and *C*_V2_, are formed between nodes 2′ and 3 and nodes 5 and 6, respectively. These variable capacitances originate from changes in the effective overlapping area during relative motion. Through nodal analysis, the circuit is further simplified by combining capacitive and resistive elements and introducing equivalent current sources, yielding the current-source equivalent model of the TENG shown in Figure S2. Based on this model, the output characteristics of the FC-TENG can be theoretically derived. Furthermore, as detailed in Methods S1, this approach is extended to derive the impedance variation and voltage superposition behavior of multiple units connected in parallel, providing an explanation for the experimentally observed nonlinear increase in *V*_OC_.

Based on nodal analysis and the current-source equivalent circuit model, the internal resistance of the FC-TENG (*R*_0_) can be expressed as shown in Equation 1.

(Equation 1) $$R_{0}=R_{\text{PTFE}1}+R_{\text{RH}1}+R_{\text{RH}2}+R_{\text{PTFE}2}\text{.}$$

Here, *R*_*PTFE1*_, *R*_*RH1*_, *R*_*RH2*_, and *R*_*PTFE2*_ denote the internal resistances arising from frictional contact between the two triboelectric materials, PTFE and RH, respectively. The internal capacitance of the FC-TENG (*C*_0_) consists of multiple capacitive components connected in series. Accordingly, *C*_0_ can be expressed as shown in Equation 2.

(Equation 2) $$C_{0}=\frac{1}{\frac{1}{C_{C1}}+\frac{1}{C_{\text{CV}1}}+\frac{1}{C_{\text{CV}2}}+\frac{1}{C_{C2}}}\text{.}$$

Based on the equivalent circuit of the flexible power-generation device and applying Kirchhoff’s laws, the output voltage (*V*_RL_) and output current (*I*_RL_) of the TENG can be expressed as Equations 3 and 4, respectively.^29^^,^^30^

(Equation 3) $$V_{\text{RL}}=\frac{I_{0}R_{0}R_{L}}{R_{0}+\sqrt{R_{L}^{2}+{\left(\frac{1}{2\pi fC_{0}}\right)}^{2}}}\text{.}$$

(Equation 4) $$I_{\text{RL}}=\frac{I_{0}R_{0}}{R_{0}+\sqrt{R_{L}^{2}+{\left(\frac{1}{2\pi fC_{0}}\right)}^{2}}}\text{.}$$

Based on the equivalent circuit of the TENG, the *V*_OC_ and *I*_SC_ under external loading conditions can be derived using limit analysis. These quantities are expressed in Equations 5 and 6, respectively.^31^^,^^32^

(Equation 5) $$V_{\text{OC},R_{L}\to \infty }=I_{0}R_{0}\text{.}$$

(Equation 6) $$I_{\text{SC},R_{L}\to \infty }=\frac{I_{0}R_{0}}{R_{0}+\frac{1}{2\pi fC_{0}}}\text{.}$$

Figure 2C presents finite-element simulation results of charge transfer within the FC-TENG, resulting in distinct voltage distributions across the interdigitated electrodes. The color scale represents the voltage magnitude, where red indicates a high voltage reaching up to 2.38 × 10^3^ V, closely matching the experimentally measured output voltage of 2,284 V. These simulation results provide theoretical guidance for enhancing the electrical performance of the FC-TENG through rational structural optimization.

### Optimization of structural parameters and their effects on output performance of the FC-TENG

**Figure 3:**
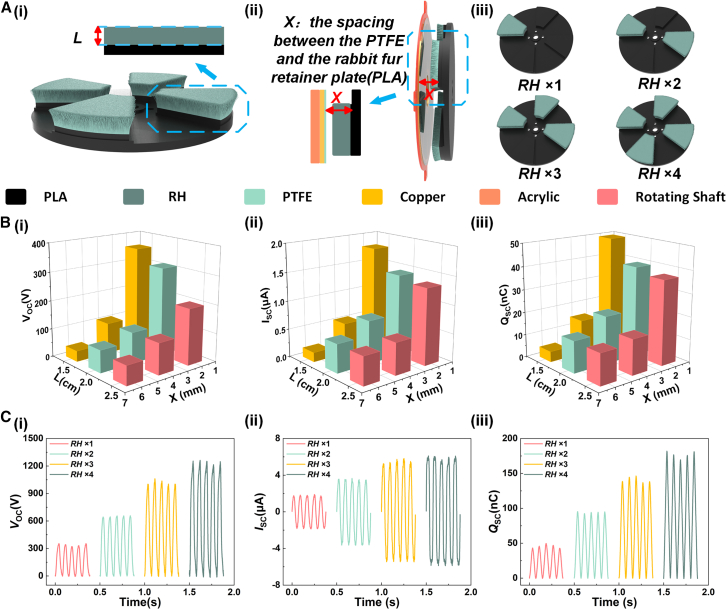
Structure parameters and output performance of the FC-TENG power-generation unit (A) Schematic diagram of the structure parameters of the FC-TENG power-generation unit. (B) Output performance under different *X* and *L*. (C) Output performance under different numbers of rabbit-hair patches.

Figure 3A illustrates the structural parameters of the FC-TENG power-generation units. Specifically, Figure 3A(i) shows the schematic definition of RH length (*L*). Three lengths were investigated, namely 1.5, 2, and 2.5 cm. Figure 3A(ii) presents the spacing (*X*) between the PTFE film (serving as the triboelectric layer) and the PLA RH carrier plate, to which the RH is firmly attached. Three spacing values were evaluated: 2, 4, and 6 mm. Figure 3A(iii) depicts the number of RH patches on a single plate, configured as *RH* × 1, *RH* × 2, *RH* × 3, and *RH* × 4. Although configurations with more than four RH patches were considered, both experimental observations and theoretical analysis indicate that the associated trade-offs outweigh the marginal performance benefits. Specifically, when the number of patches exceeds four, spatial constraints within the compact structure lead to overlap between adjacent units, reducing the effective frictional contact area and resulting in diminishing output gains.

In addition, increasing the number of patches requires higher manufacturing precision, which raises fabrication complexity and cost and is therefore inconsistent with the low-cost requirements of railway-side applications. It also increases the internal capacitance (Equation 2), which may disrupt impedance matching with external loads such as railway monitoring sensors. Furthermore, greater structural loading introduces higher rotational mechanical stress, increasing the risk of fiber entanglement, non-uniform wear, and reduced device durability. Accordingly, the *RH* × 4 configuration provides an optimal balance among output performance, manufacturability, impedance matching, and mechanical reliability, thereby meeting the requirements of railway-based wind energy harvesting and self-powered systems.^6^ In addition, all materials used in the structural design are clearly identified, including PLA, RH, PTFE, copper, acrylic, and the rotating shaft.

Figures 3B and S5 present the *V*_OC_, *I*_SC_, and short-circuit charge of a single standardized RH patch (*RH* ***×*** 1) under different combinations of *X* and *L*. These results are used to determine the optimal structural configuration of the device. The experimental results indicate that, at ***X*** = 2 mm and *L* = 1.5 cm, *RH* ***×*** 1 exhibits the best overall output performance, with a peak *V*_OC_ of 358 V, a peak *I*_SC_ of 1.8 μA, and a peak *Q*_SC_ of 49.5 nC. This behavior can be attributed to two main factors. First, during rotation, the RH fibers can fully extend and conform to the frictional interface, ensuring efficient triboelectrification and significantly enhancing charge accumulation at the contact surface. Second, at this length, the RH structure exhibits favorable mechanical stability, effectively preventing fiber breakage or entanglement associated with excessive length. This helps maintain a stable contact frequency and frictional state, thereby avoiding fluctuations in charge generation caused by structural instability. In contrast, when *L* is relatively large and *X* is increased, the RH fibers are constrained by neighboring fibers and cannot fully extend toward the frictional interface. As a result, the number of fibers actively participating in triboelectrification is significantly reduced.

On this basis, Figure 3C further investigates the influence of the number of RH patches on a single plate (*RH* × 1, *RH* × 2, *RH* × 3, and *RH* × 4) on the output performance of the FC-TENG. The results show that as the number of RH patches increases from *RH* × 1 to *RH* × 4, the peak values of *V*_OC_, *I*_SC_, and *Q*_SC_ increase progressively. Specifically, under the *RH* × 4 configuration, *V*_OC_ reaches 1,250 V, *I*_SC_ reaches 6 μA, and *Q*_SC_ reaches 180 nC. This improvement can be attributed to the increased number of RH patches, which enhances the effective contact area and contact frequency at the friction interface. In addition, the cooperative interaction among multiple patches strengthens the triboelectrification and charge transfer processes, leading to improved electrical output. Accordingly, the optimized structural parameters for a single power-generation unit (1U) are determined as *X* = 2 mm, *L* = 1.5 cm, and *RH* × 4 for the number of RH patches on a single plate.

### Performance characterization, load matching, and durability analysis of a single power-generation unit

**Figure 4:**
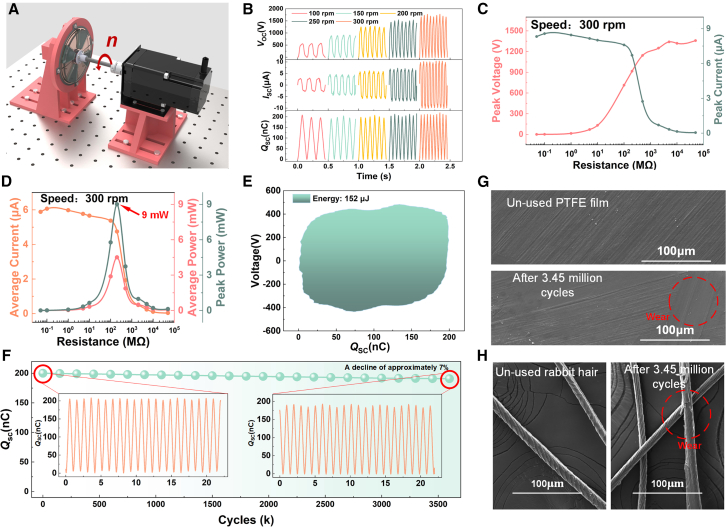
Output performance and durability test of a single power-generation unit (A) Experimental platform of a single FC-TENG power-generation unit. (B) Performance output at different rotational speeds. (C) Peak voltage and peak current variation with load at 300 rpm. (D) Output power variation with load resistance at 300 rpm. (E) Single energy at 200 MΩ load. (F) Cycling stability test of transferred charge. (G) The changes in the surface morphology of PTFE material before and after wear. Scale bars: 100 μm. (H) The surface morphology changes of RH. Scale bars: 100 μm.

After determining the optimal structural parameters of a single FC-TENG power-generation unit, performance characterization was carried out using the experimental platform shown in Figure 4A. The effects of rotational speed (100–300 rpm) on the *V*_OC_, *I*_SC_, and *Q*_SC_ of 1U were systematically investigated, as presented in Figure 4B. The results indicate a strong positive correlation between rotational speed and the peak values of *V*_OC_ and *I*_SC_. In contrast, the peak value of *Q*_SC_ remains relatively stable with increasing rotational speed. This behavior can be attributed to the fact that *Q*_SC_ is primarily governed by the intrinsic material electronegativity difference, effective contact area, and device structural parameters, rather than dynamic operating conditions.

Furthermore, under the optimal rotational speed of 300 rpm, load tests were conducted on the single power-generation unit of the FC-TENG. Figure 4C illustrates the variation in peak voltage and peak current as the load resistance increases from 0.05 to 5 × 10^4^ MΩ. The peak voltage gradually increases and tends to saturate with rising load resistance. When the load resistance reaches 5 × 10^4^ MΩ, the peak voltage approaches 1,360 V. This behavior can be explained by the formation of an external circuit through the load resistance, which allows current flow and induces a voltage drop across the internal resistance (R_0_) of the TENG. Consequently, the voltage measured at the load is lower than the *V*_OC_ under identical operating conditions. In contrast, the peak current decreases exponentially with increasing load resistance, dropping from 8.57 μA at 0.1 MΩ to 0.049 μA at 5 × 10^4^ MΩ, which is consistent with Ohm’s law.

Based on the peak current, average current, and load resistance data, the output power is calculated using *P* = *I*^2^*R*. As shown in Figure 4D, both peak and average power exhibit a unimodal (single-peak) dependence on load resistance. The maximum effective power of 4.5 mW is achieved at a load resistance of 200 MΩ. Figure 4E presents the Lissajous curve of voltage versus *Q*_SC_ at a load resistance of 200 MΩ. The energy output per cycle is obtained by integrating the enclosed area of this curve, yielding a single-cycle energy transfer of 152 μJ. Figure 4F shows the durability performance of 1U. After 3.45 million cycles, the *Q*_SC_ decreases from an initial value of approximately 200.58 nC to about 185.61 nC, corresponding to a degradation rate of only 7%. The insets present representative charge output curves at the initial stage and after long-term cycling.

Figure 4G illustrates the surface morphology evolution of PTFE before and after testing. As the primary triboelectric layer, PTFE exhibits only minor surface scratches after 3.45 million cycles, with no significant peeling or deformation. This confirms that the RH-based flexible contact design effectively mitigates mechanical wear, ensuring stable long-term performance. Figure 4H shows the corresponding surface changes of RH before and after wear testing. After 3.45 million cycles, slight wear is observed on individual fibers (highlighted by the red dashed box in Figure 4H), while the overall structural integrity remains intact. These results confirm that structural parameter optimization effectively suppresses performance degradation caused by severe fiber wear or fracture, thereby maintaining a stable contact interface.

Additional SEM images of PTFE, RH, and copper electrodes are provided in Figure S6. After long-term operation, PTFE shows only minor surface abrasion, and RH exhibits slight localized wear, while both materials retain their functional integrity. This results in an ultra-low performance degradation rate (7%), effectively addressing the wear limitations of rigid-contact TENGs and supporting the long-term stable operation of FC-TENGs in harsh railway environments. Overall, these findings highlight the advantages of the proposed system for outdoor distributed self-powered applications.

### Output characteristics and power optimization analysis of multi-power-generation units in parallel connection

**Figure 5:**
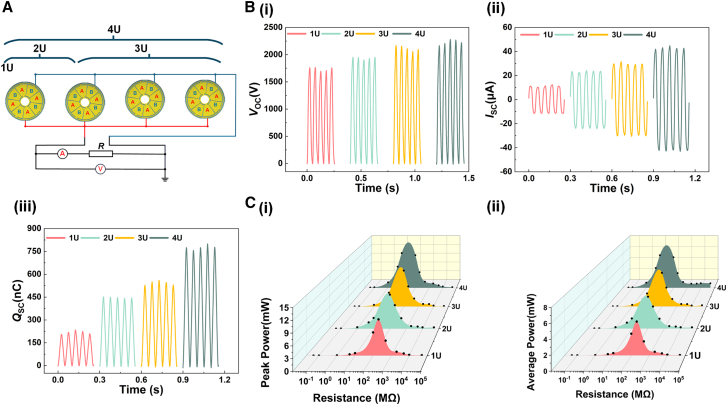
Performance of multi-unit parallel output (A) Multi-unit parallel integration of the FC-TENG. (B) Performance output with different numbers of parallel units. (C) Peak power and average power-load resistance characteristics with different numbers of parallel units.

Figure 5A presents a schematic of the parallel integration of one to four power-generation units with optimized structural parameters. A co-phase design is adopted to ensure synchronous superposition of the output signals from each unit, and the units are interconnected in parallel via external wiring. Figure 5B shows the output characteristics of *V*_OC_, *I*_SC_, and *Q*_SC_ for configurations ranging from 1U to 4U at a rotational speed of 300 rpm. Compared with 1U, the *V*_OC_ of 4U increases to 2,284 V, the *I*_SC_ rises to 86.48 μA (3.7 times that of 1U), and the *Q*_SC_ reaches 794 nC (3.8 times that of 1U). These results are consistent with the typical parallel behavior of TENGs, in which current and charge approximately scale linearly with the number of units.

When multiple units are connected in parallel (with the equivalent current-source circuit models for a single unit and four parallel units shown in Figures S2–S7, respectively), the *V*_OC_ exhibits a moderate increase. This is because, in practical measurements, the observed *V*_OC_ corresponds to the potential across the internal resistance of the electrometer (*V*_RL_), rather than an ideal infinite-impedance open circuit. Therefore, the measured voltage is jointly influenced by both internal and external resistances. Upon parallel connection, the internal resistances of individual units are effectively connected in parallel, while their internal capacitances are arranged in series. This combined effect reduces the overall internal resistance of the FC-TENG, whereas the external load resistance (*R*_L_) remains unchanged. According to the voltage division principle, the voltage across the external load increases accordingly. However, since the internal resistance of the TENG is much smaller than that of the electrometer (*R*_L_), *V*_RL_ increases only moderately rather than proportionally after parallel connection. A detailed derivation is provided in Methods S1.^33^

Figures 5C, S8, and S9 present the peak and average power curves of 1U–4U under varying load resistances. All configurations exhibit a single-peak dependence of power on load resistance. The optimal load resistance ranges from 10 to 200 MΩ, which is largely consistent with the optimal value of 1U (200 MΩ). When four units are connected in parallel, the peak output power reaches 10.68 mW. Overall, the parallel-integrated FC-TENG system not only maintains high-voltage output and stable operation but also enables efficient scaling of output performance through modular integration.

### Practical application verification of the FC-TENG in railway scenarios

**Figure 6:**
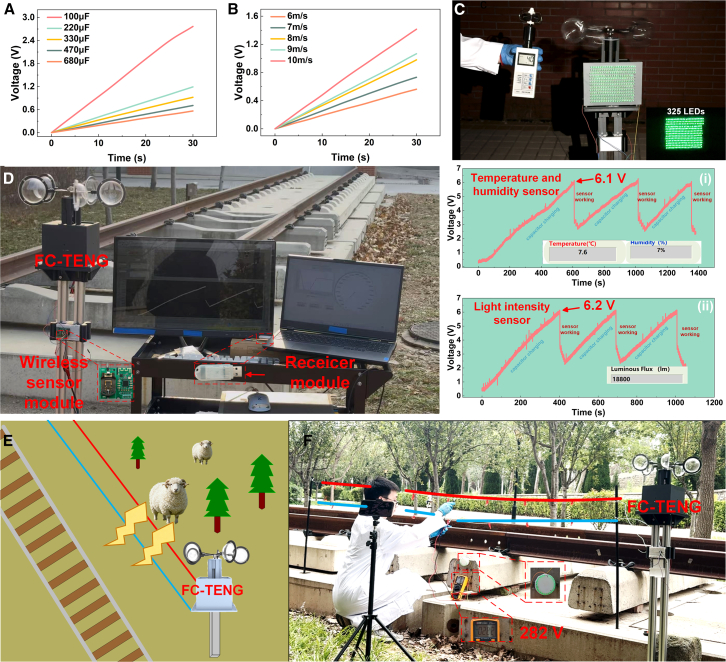
Applications of the FC-TENG (A) Charging characteristics of different capacitances. (B) Charging characteristics at different wind speeds. (C) Physical picture of the FC-TENG driving an LED array. (D) On-site picture of the self-powered environmental monitoring system for railways. (E) Schematic of the active protection wire system for railways. (F) Experimental diagram of the active protection wire system for railways.

To evaluate the wind energy storage performance and capacitance adaptability of the FC-TENG, Figure 6A presents the voltage-time charging curves of a prototype equipped with wind cups designed for efficient wind energy capture. Measurements were conducted at a constant wind speed of 7 m/s using capacitors with different capacitances (100, 220, 330, 470, and 680 μF). The results show that, for a 100 μF capacitor, the voltage rapidly increases to 2.8 V within 30 s. Figure 6B further illustrates the charging behavior of a 470 μF capacitor under varying wind speeds. At a wind speed of 10 m/s, the voltage rises quickly to 1.5 V within 30 s. As shown in Figure 6C, under a wind speed of approximately 5 m/s, typical of railway embankment environments, the device is capable of illuminating 325 LEDs (Video S1).

Video S1. FC-TENG power supply for lights

Figure 6D presents the application performance of the self-powered environmental monitoring system developed based on the FC-TENG. In this configuration, the system operates in a “charge-operation” cyclic power-supply mode. At a wind speed of approximately 5 m/s, the FC-TENG charges a 940 μF capacitor through a rectifier bridge. Once the capacitor voltage reaches a predefined threshold, it immediately releases stored energy to drive the sensor. During this operation phase, the sensor functions normally, collects environmental data, and transmits the information wirelessly. As the temperature and humidity sensor (Figure 6D[i]; Video S2) and the light intensity sensor (Figure 6D[ii]; Video S3) consume energy, the capacitor voltage gradually decreases. When it falls below the preset threshold, the sensor automatically stops operating, and the FC-TENG resumes charging the capacitor, initiating the next operational cycle. Figures 6E and 6F propose a railway active protection wire system powered by the FC-TENG. The FC-TENG is electrically connected to the protective wire via conductive leads. At a wind speed of approximately 5 m/s, the system outputs a voltage of around 300 V to the wire, which is demonstrated by lighting 25 LEDs (Video S4). This voltage level can serve as an effective warning signal for approaching individuals. When a person or animal approaches or contacts the protective wire, a non-lethal but clearly perceptible electric stimulus is generated, prompting an immediate avoidance response.

Video S2. FC-TENG power supply for temperature and humidity sensor

Video S3. FC-TENG power supply for luminous flux

Video S4. FC-TENG power supply for railway safety protection

## Results

This study proposes a high-performance FC-TENG based on RH for wind energy harvesting along railway lines. By employing RH as a flexible electrode and optimizing its structural parameters, the issues of underutilized performance and insufficient durability in RH-based electrodes are effectively addressed, leading to significant improvements in both output performance and long-term stability. The FC-TENG was systematically developed and evaluated through nodal analysis, finite-element simulations, and comprehensive electrical performance testing. The experimental results show that, under identical operating conditions, parallel connection of multiple power-generation units increases the current by a factor of 3.7 and the *Q*_SC_ by a factor of 3.8 compared with a single unit. The maximum output power reaches 10.68 mW. In addition, after 3.45 million continuous operating cycles, the single-unit device exhibits only a 7% degradation in output performance, demonstrating excellent durability. Furthermore, the FC-TENG was validated in multiple application scenarios, including sensor power supply, wireless environmental monitoring, and active safety protection systems. The results confirm its capability to reliably power electronic devices and support environmental monitoring along railway lines, while also enabling active safety protection functions. Overall, the proposed system extends the application scope of RH-based FC-TENGs in railway wind energy harvesting and enables stable power supply for low-power devices. This work not only broadens the application potential of RH in flexible TENG systems but also provides a valuable reference for the development of self-powered intelligent monitoring and protection systems in railway infrastructure.

### Experimental section

#### Fabrication of the FC-TENG

The FC-TENG is primarily composed of a transmission unit, a TENG power-generation unit, and a supporting structure. The transmission unit consists of a transmission shaft, wind cups, and a flange coupling. The transmission shaft has a diameter of 6 mm and a total length of 350 mm. The wind cups are hemispherical acrylic components with a diameter of 70 mm. Five wind cups are uniformly arranged in a circumferential configuration, forming an overall wind cup assembly diameter of 300 mm. The assembly is fixed to one end of the transmission shaft via a flange coupling. The TENG power-generation unit comprises interdigitated electrodes fabricated from copper foil deposited on an acrylic substrate, together with PTFE and RH as triboelectric materials. The outer diameter of the interdigitated electrodes is 70 mm, and the acrylic substrate has a thickness of 3 mm. A PTFE film is placed above the interdigitated electrode layer. Four standardized RH patches (*RH* ***×*** 4) are mounted on a circular rotor with an outer diameter of 84 mm, which is fabricated using PLA via 3D printing. The supporting housing is also fabricated from PLA using 3D printing. The overall dimensions of the FC-TENG are 150 mm (length) × 130 mm (width) × 300 mm (height). Four power-generation units are evenly distributed and securely installed within the housing. In addition, two bearings with an inner diameter of 6 mm are mounted at both ends of the structure to ensure stable rotation. The RH used in this study is a standardized raw material supplied by Xinxiang Rongtai Animal Fiber Co., Ltd. (model RT-WH-001). Prior to device fabrication, the RH undergoes uniform surface treatment, including chrome salt tanning and dry-cleaning purification. These processes are commonly used in commercial RH processing to effectively remove surface impurities (e.g., dust, oils, and residual moisture), maintain dryness, and preserve its intrinsic microstructure and triboelectric properties.

#### Experimental process and measuring equipment

The experimental setup (25°C, 50% RH) consists of a rotating motor system and a data acquisition system. The rotating motor (model: 57BYG250H-8 PFDE, China) is securely mounted on an optical platform, and its output shaft is coupled to the transmission shaft of the power-generation unit to drive rotor rotation. An external airflow source is provided by a blower (model: DL661400, Deli, China), while wind speed is measured using an anemometer (model: UT363S, UNI-T, China). The data acquisition system includes a data acquisition card (NI USB-6009, USA), an electrometer (Keithley 6514, USA), and a computer. The electrometer is used to measure the electrical output signals of the prototype, which are then transmitted to the computer via the data acquisition card. The acquired signals are subsequently recorded and processed using LabVIEW software. Surface morphology and wear characteristics of the friction layers were analyzed using a scanning electron microscope (Quanta 450 FEG, Thermo Fisher Scientific, Germany).

### Limitations of the study

This work develops an RH-based FC-TENG and demonstrates its feasibility for railway line monitoring and safety protection through systematic theoretical analysis and experimental validation, providing a useful reference for self-powered railway monitoring systems. However, several aspects of this study still require further improvement and investigation. First, the wind energy harvesting performance of the FC-TENG is primarily evaluated under controlled laboratory conditions with stable wind speeds, while its operational behavior under natural, dynamically varying wind conditions remains to be systematically verified in future studies. Second, the current power management system is relatively basic, relying on a simple rectifier-capacitor-based cyclic charging mode, and therefore, the energy management efficiency and intelligent regulation capability require further optimization. In addition, the prototype fabrication of the FC-TENG largely depends on manual assembly and 3D printing techniques. The lack of standardized, scalable manufacturing processes for key components remains a limitation, which should be addressed to better meet the requirements of low-cost, distributed railway monitoring applications.

## Resource availability

### Lead contact

Requests for further information and resources should be directed to and will be fulfilled by the lead contact, Da Zhao (zhao_da@yeah.net).

### Materials availability

This study did not generate new unique reagents.

### Data and code availability

• Partial data reported in this article will be shared by the lead contact upon request.
• This article does not report original code.
• Any additional information required to reanalyze the data reported in this article is available from the lead contact upon request.

## Acknowledgments

The authors are grateful for the support received from the Key Project from Shandong Province Intelligent Equipment Innovation and Entrepreneurship Community for Construction Machinery (no. GTT20240105), Key R&D Program of Shandong Province, China (nos. 2025JMRH0304 and 2023CXGC010206), and the Undergraduate Teaching Reform Research Project of Shandong Jiaotong University (no. 2023YB19).

## Author contributions

L.C.: conceptualization, investigation, validation, writing – original draft, and writing – review and editing; L.Z.: supervision, conceptualization, and writing – review and editing; S.W.: writing – review and editing, investigation, and validation; B.W.: writing – review and editing and investigation; Y.Q.: writing – review and editing and investigation; D.Z.: writing – review and editing, resources, and supervision.

## Declaration of interests

The authors declare no competing interests.

## STAR★Methods

### Key resources table

**Table:** 

REAGENT or RESOURCE	SOURCE	IDENTIFIER
**Software and algorithms**		

NI LabVIEW 2024 Q1	National Instruments	Version 2024 Q1 (32-bit)
SolidWorks 2024	Dassault Systemes	Version 2024 SP0
Origin Pro 2022	OriginLab Corporation	Version 2022
Visio 2024	Microsoft Corporation	Version 2024
Keyshot 11	Luxion Corporation	Version 11

**Other**		

Rabbit hair	Xinxiang Rongtai Animal Fiber Co., Ltd. (China)	Model:RT-WH-001
PTFE	Zhongxing Huacheng Co., Ltd. (Japan/China)	Model:ASF-110FR
Copper foil	Shandong Jiaotong University	N/A
PLA filament (3D printing)	Bambu Lab (China)	Model:Basic PLA
Acrylic plate	Jinyi Organic Glass Products Factory (China)	Model:3 mm
Bearing	XUDZ (Japan)	Model:626ZZ
Transmission shaft	Shandong Jiaotong University	N/A
Flange coupling	Shandong Jiaotong University	N/A
3D printer	Bambu Lab (China)	Model:A1
Rotating motor	Standard motor manufacturer (China)	Model:57BYG250H-8 PFDE
Blower	Deli Group Co., Ltd. (China)	Model:DL661400
Anemometer	UNI-T Electronics Co., Ltd. (China)	Model:UT363S
Electrometer	Keithley Instruments, LLC (USA)	Model 6514
Scanning electron microscope (SEM)	Thermo Fisher Scientific (Germany)	Model:QuantaTM 450 PEG
Data acquisition card	National Instruments (NI) (USA)	Model:USB-6009
Optical platform	Cangzhou Yihang Technology Co., Ltd. (China)	Model:1200 × 1500 mm

### Method details

#### Power generation units fabrication and assembly

The power-generation unit consists of a stator and a rotor. The stator is composed of an acrylic substrate, interdigitated copper electrodes fabricated on the acrylic substrate, and a PTFE film deposited over the electrode surface. The rotor comprises a rabbit-hair-bearing plate and rabbit-hair patches.

The acrylic substrate was prepared from a 3 mm acrylic sheet, which was cut into the desired geometry using a laser cutter. The engraving function of the laser cutter was further used to define electrode-pattern grooves with a depth of 1 mm on the acrylic substrate. Subsequently, copper foil was laminated onto the substrate and manually cut along the predefined grooves using a carving knife, after which the excess copper foil was removed, yielding well-defined interdigitated copper electrodes firmly adhered to the acrylic base. Finally, a PTFE film was deposited over the entire electrode region, serving as the triboelectric layer on top of the interdigitated electrodes.

The rabbit-hair-bearing plate of the rotor was fabricated via 3D printing, while the rabbit-hair patches were prepared by cutting raw RH into uniform segments.

#### Electrical characterization setup

The electrical characterization setup of the FC-TENG consists of a mechanical drive unit, a wind supply unit, a high-precision signal acquisition system, and a data processing unit. All experiments were conducted at a controlled temperature of 25 °C and a relative humidity of 50% RH to minimize environmental interference. A stepping motor (57BYG250H-8 PFDE) mounted on a 1200 × 1500 mm optical platform was used to drive the rotation of the FC-TENG through a flange coupling, with the rotational speed adjustable from 100 to 300 rpm. A DL661400 blower provided the airflow source, while a UT363S anemometer was used to monitor the wind speed at the position of the wind cups in real time. Electrical outputs, including open-circuit voltage (*V*_OC_), short-circuit current (*I*_SC_), and transferred charge (*Q*_SC_), were measured using a Keithley 6514 electrometer. The analog signals were converted into digital signals via an NI USB-6009 data acquisition card and transmitted to a computer. NI LabVIEW 2024 Q1 software was used for real-time signal acquisition and preliminary filtering, and OriginPro 2022 was employed for subsequent data analysis, curve fitting, and performance parameter calculations. For load-matching experiments, precision resistors ranging from 0.05 MΩ to 5 × 10^4^ MΩ were used as external loads to evaluate voltage and current responses and determine the optimal load resistance. Durability tests were conducted at a constant rotational speed of 300 rpm, during which the transferred charge was continuously recorded over 3.45 million cycles to assess long-term performance degradation.

### Quantification and statistical analysis

#### Data analysis and processing

For the output performance evaluation of the FC-TENG single power-generation unit and multi-unit parallel system, an electrometer was used to perform three repeated measurements of open-circuit voltage (*V*_OC_), short-circuit current (*I*_SC_), and transferred charge (*Q*_SC_) under different rotational speeds and load resistance conditions. The averaged values were taken as the final results to minimize random experimental errors. The peak output power of the FC-TENG was calculated using *P*=*I*^2^*R* based on the measured current and load resistance. The single-cycle energy output was obtained by integrating the area enclosed by the voltage– *Q*_SC_ Lissajous curve. For durability testing, the transferred charge of a single power-generation unit was continuously recorded over 3.45 million operating cycles, and the performance degradation rate was determined by comparing the initial and post-cycling charge values. The surface morphologies of the friction materials before and after testing were characterized using scanning electron microscopy (SEM), and wear behavior was evaluated by analyzing structural changes in the RH and PTFE surfaces.

#### Explanation of the number of core repetitions

All experiments were conducted with at least three independent replicates under identical conditions (including wind speed, device configuration, ambient temperature, and relative humidity) to ensure the reliability and reproducibility of the results, except for the durability test, which was performed once. For the output performance measurements (including open-circuit voltage, short-circuit current, output power, and transferred charge), the reported values correspond to the maximum values obtained from valid replicates after excluding abnormal outliers.

#### Software

Finite element simulations of the interdigital electrode voltage distribution in the FC-TENG were performed using COMSOL Multiphysics 6.3. Real-time acquisition, recording, and preliminary filtering of electrical output signals were implemented using NI LabVIEW 2024 Q1. Experimental data processing, including curve plotting, parameter calculation, and curve fitting, was conducted in OriginPro 2022. The structural design and three-dimensional modeling of the FC-TENG prototype were carried out using SolidWorks 2024, while schematic diagrams and layout optimization were completed using Microsoft Visio 2024.

## Supplemental information

Document S1. Figures S1–S9 and Method S1
