# Supplementary material for: Wind energy-driven rabbit hair TENG for railway detection
Source: iScience. 2026 Jun 11;29(7):116335. doi: 10.1016/j.isci.2026.116335 (PMC13276440; doi:10.1016/j.isci.2026.116335)
Supplement: Document S1. Figures S1–S9 and Method S1 [file mmc1.pdf]

iScience, Volume *29*

Supplemental information

Wind energy-driven rabbit hair TENG for railway detection

Linchao Chen, Lin Zhang, Shipeng Wang, Baoping Wang, Yubin Qi, and Da Zhao

Supporting Information

Supporting Figures

Figure S1 Actual structure of the prototype, Related to Figure 1

Figure S2 The equivalent circuit model of FC-TENG, Related to Figure 2.

Figure S3 Comparison of short-circuit current with the performance of other flexible contact TENGs, Related to Figure 1.

Figure S4 Comparison of transferred charge with the performance of other flexible contact TENGs, Related to Figure 1.

Figure S5 Output performance of power-generation units under different ***L*** and ***X***, Related to Figure 3.

Figure S6 SEM images of surface morphologies of PTFE, rabbit hair, and copper electrode before and after 3.45 million cycles of wear, Related to Figure 4.

Figure S7 Equivalent circuit diagram of parallel connection of power generation units, Related to Figure 5.

Figure S8 The V_{OC} after parallel connection of different power generation units, Related to Figure 5.

Figure S9 The I_{SC} after parallel connection of different power generation units, Related to Figure 5.

Supporting Methods S1

Supporting Methods S1 Supplementary Explanation for the Open-Circuit Voltage Enhancement in Parallel Connection, Related to Figure 5.

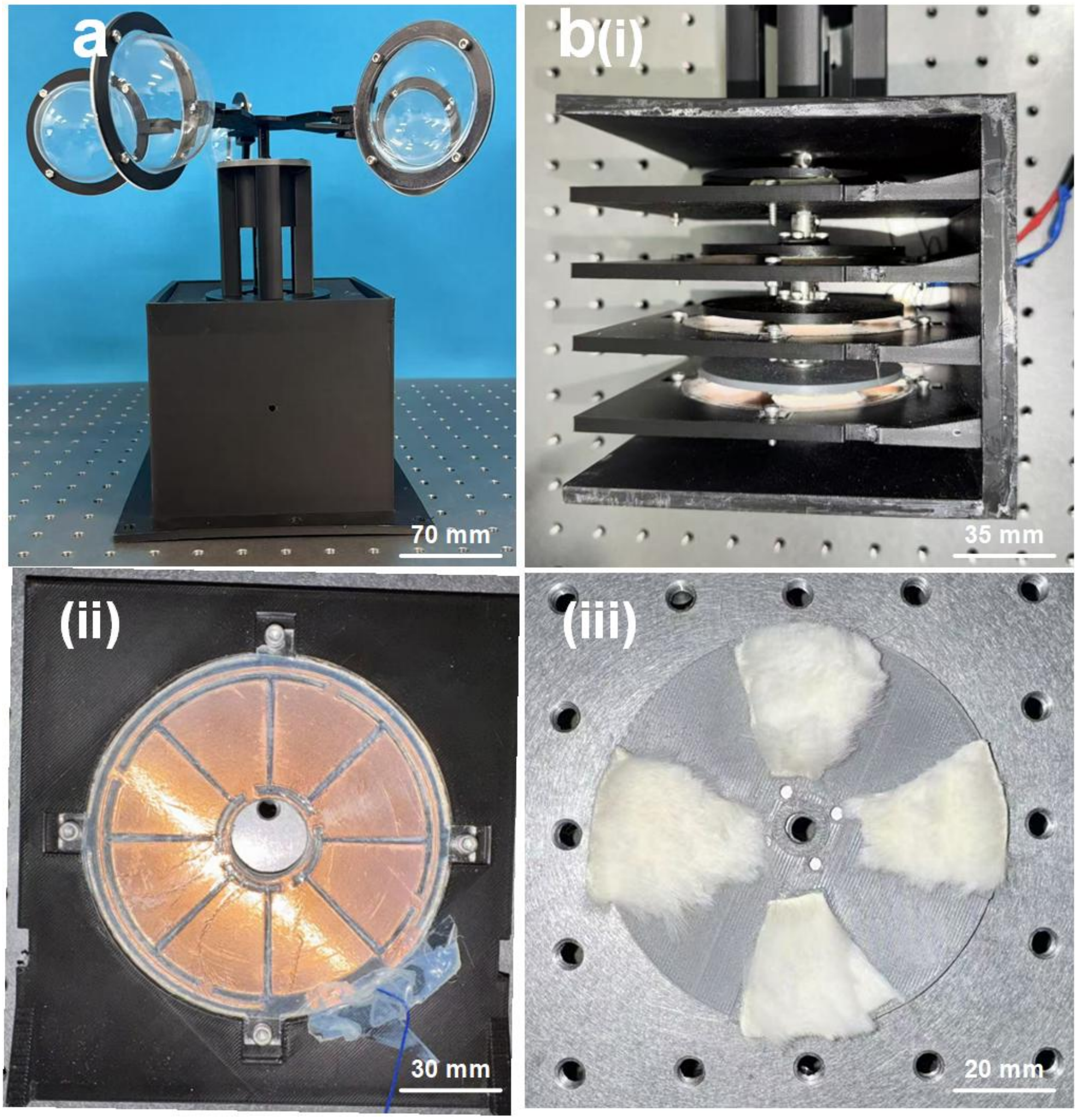

Figure S1 Actual structure of the prototype, Related to Figure 1.

(a) Whole prototype.Scale bar: 70 mm.

(b) Power generation unit: Four generating units are connected in parallel.Scale bar: 35 mm (i), Copper electrode.Scale bar: 30 mm (ii), The structure of rabbit hair.Scale bar: 20 mm (iii).

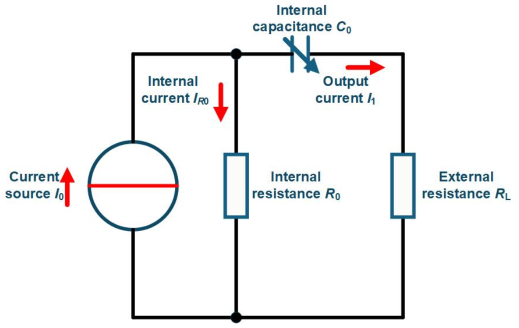

Figure S2 The equivalent circuit model of FC-TENG, Related to Figure 2.

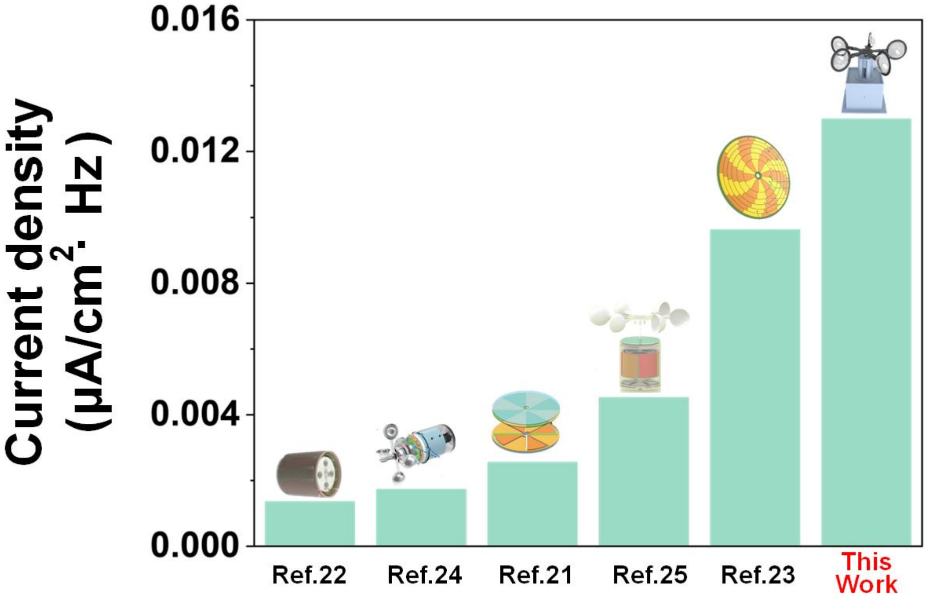

Figure S3 Comparison of short-circuit current with the performance of other flexible contact TENGs, Related to Figure 1.

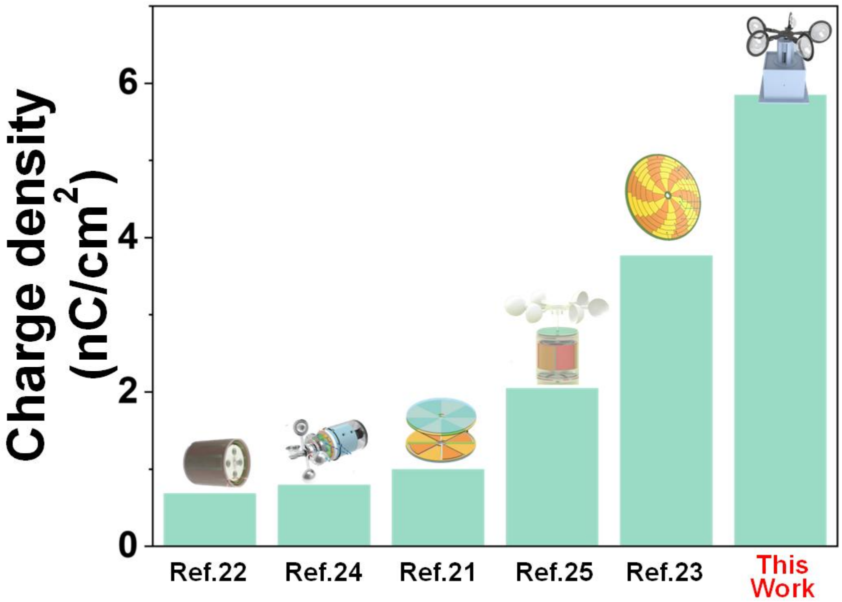

Figure S4 Comparison of transferred charge with the performance of other flexible contact TENGs, Related to Figure 1.

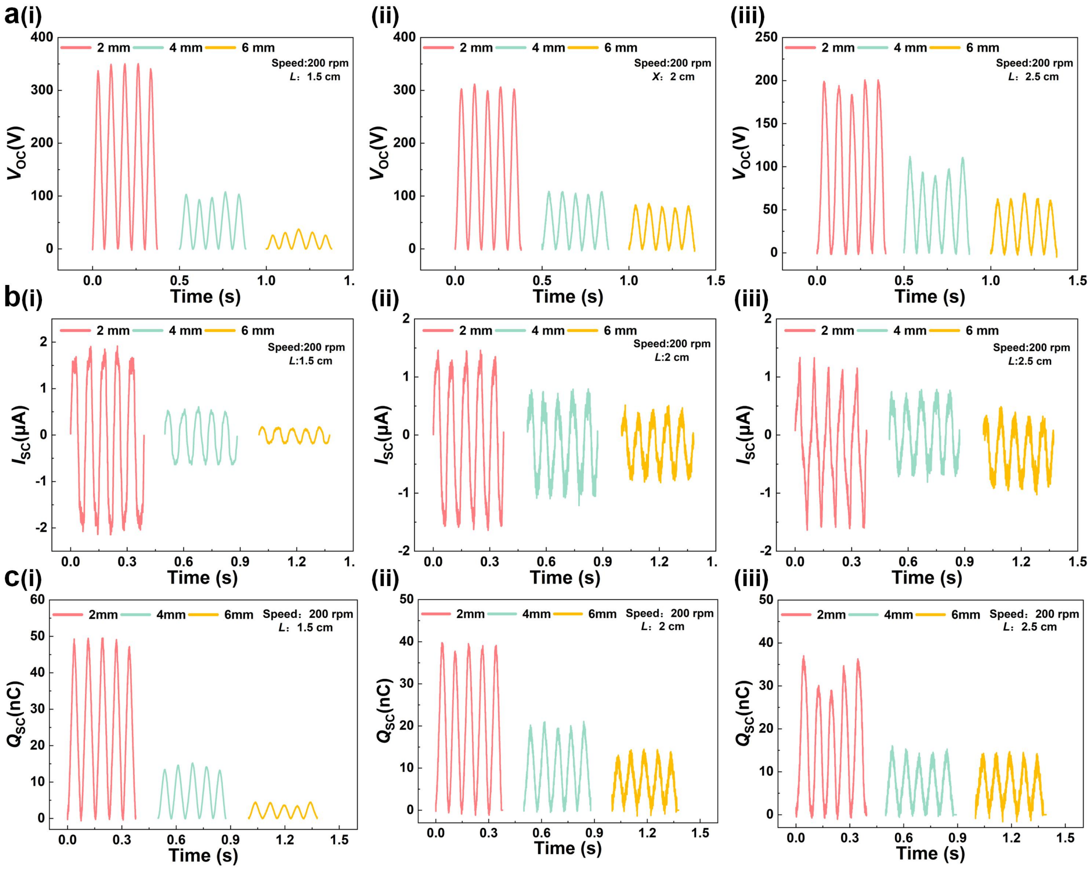

Figure S5 Output performance of power generation units under different ***L*** and ***X***, Related to Figure 3.

(a) V_{OC} under the same ***L*** but different ***X***: **L** = 1.5 cm (i), **L** = 2 cm (ii), **L** = 2.5 cm (iii).

(b) I_{SC} under the same ***L*** but different ***X***: **L** = 1.5 cm (i), **L** = 2 cm (ii), **L** = 2.5 cm (iii).

(c) Q_{SC} under the same ***L*** but different ***X***: **L** = 1.5 cm (i), **L** = 2 cm (ii), **L** = 2.5 cm (iii).

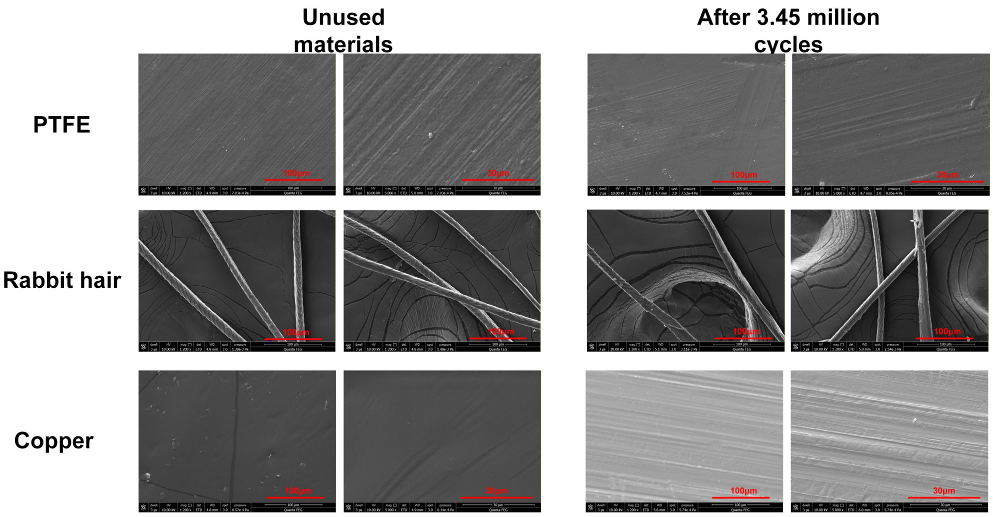

Figure S6 SEM images of surface morphologies of PTFE, rabbit hair, and copper electrode before and after 3.45 million cycles of wear.Scale bars: 100 μm for PTFE and copper electrode low-magnification images; 30 μm for PTFE and copper electrode high-magnification images; 100 μm for all rabbit hair images. (The two left-hand columns present SEM images of unused PTFE, rabbit hair, and copper, whereas the two right-hand columns display SEM images after 3.45 million cycles.), Related to Figure 4.

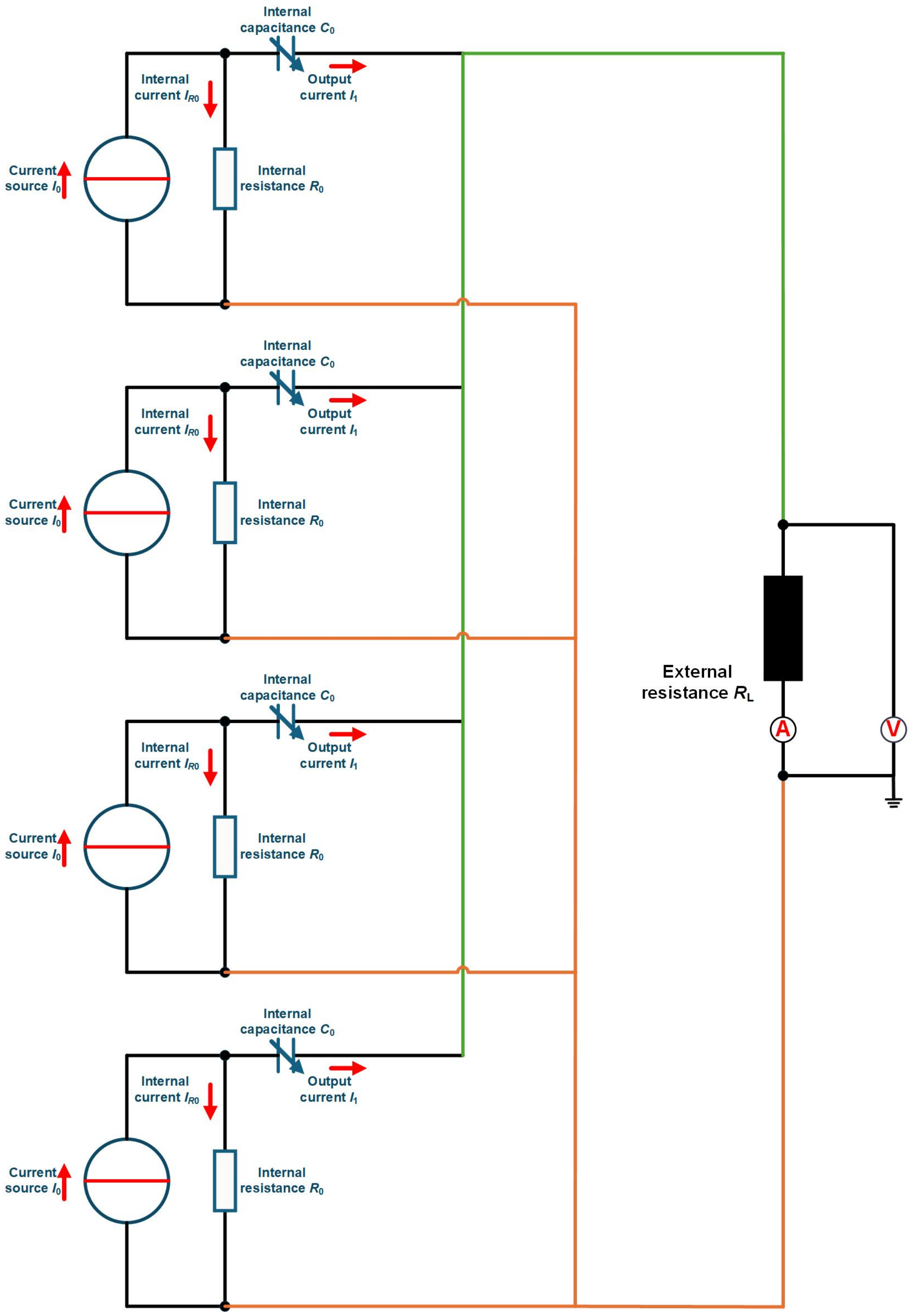

Figure S7 Equivalent circuit diagram of parallel connection of power generation units, Related to Figure 5.

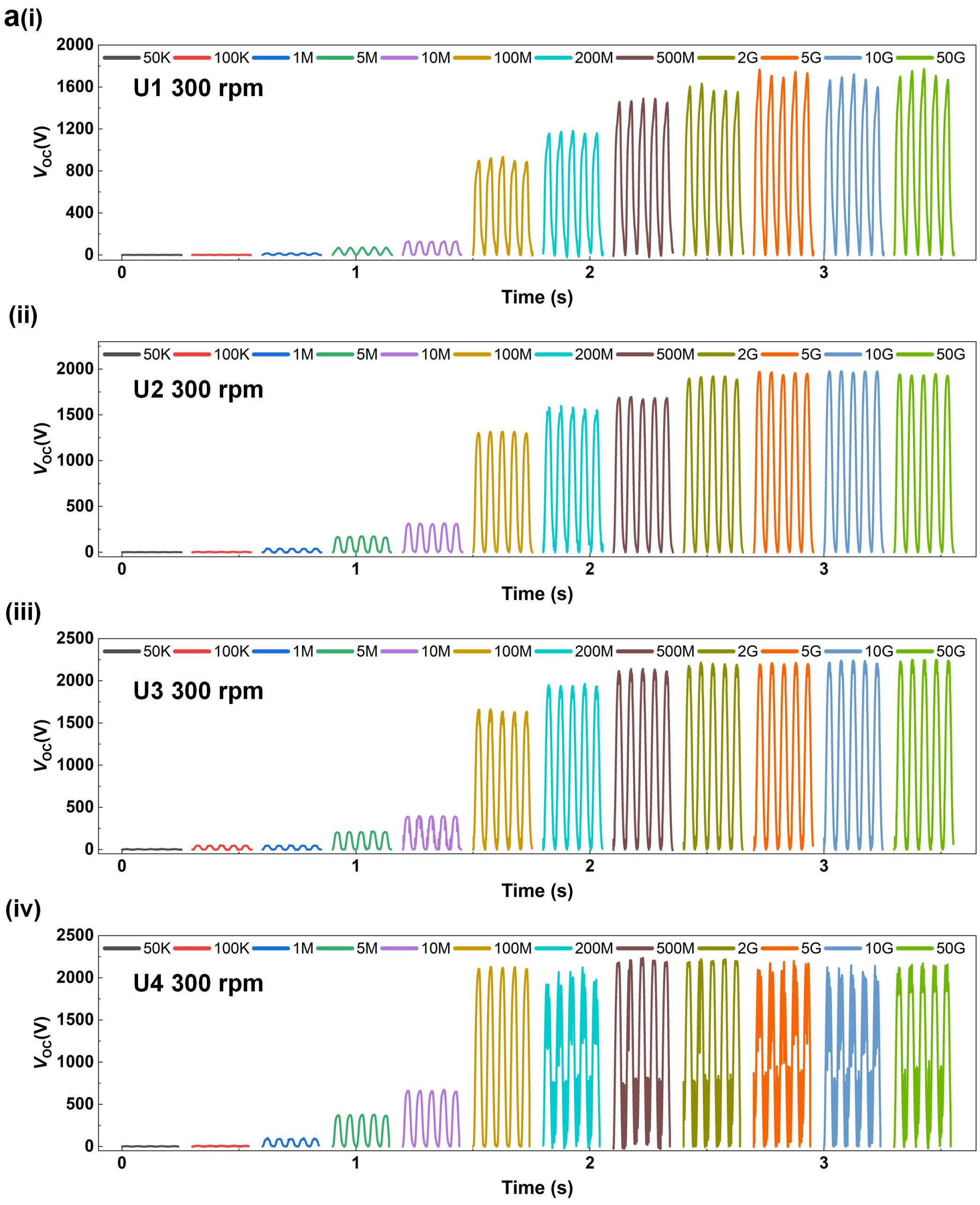

Figure S8 The V_{OC} after parallel connection of different power-generation units: The voltage of the power generation unit configured in U1 at 300 rpm (i), The voltage of the power generation unit configured in U2 at 300 rpm (ii), The voltage of the power generation unit configured in U3 at 300 rpm (iii), The voltage of the power generation unit configured in U4 at 300 rpm(iv), Related to Figure 5..

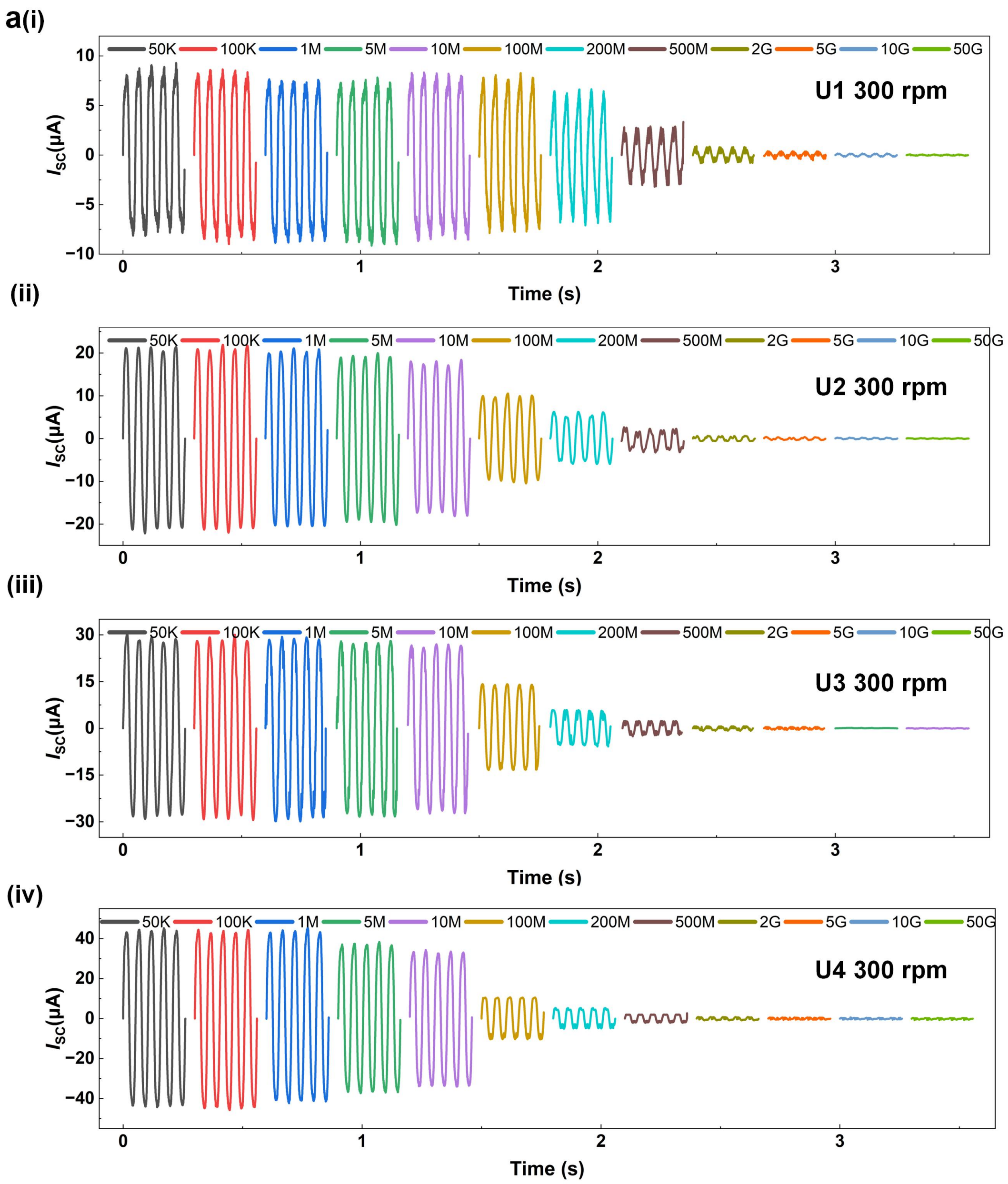

Figure S9 The I_{SC} after parallel connection of different power-generation units: The current of the power generation unit configured in U1 at 300 rpm (i), The current of the power generation unit configured in U2 at 300 rpm (ii), The current of the power generation unit configured in U3 at 300 rpm (iii), The current of the power generation unit configured in U4 at 300 rpm (iv), Related to Figure 5..

Supporting Methods S1 Supplementary Explanation for the Open-Circuit Voltage Enhancement in Parallel Connection, Related to Figure 5.

When the power generation units are connected in parallel, the open-circuit voltage exhibits a moderate increase. This is because in actual open-circuit voltage measurements, the voltage detected is the potential across the internal resistance of the electrometer (V_{RL}), rather than the ideal open-circuit condition with impedance approaching infinity. Thus, the measured open-circuit voltage is jointly influenced by both internal and external resistances. After parallel connection, the internal resistance of the FC-TENG decreases while the external resistance R_L remains constant; according to the voltage division principle, the voltage across the external load increases accordingly. However, the internal resistance of the TENG is far smaller than the internal resistance R_L of the electrometer. As a result, V_{RL} only increases moderately rather than multiplicatively after parallel connection. The specific theoretical derivation is as follows:

Based on Kirchhoff's Current Law (KCL) and the current-source equivalent circuit model of the TENG, the internal circuit current I_0 of the TENG is the sum of the internal resistance load current I_{R0} and the external branch current I_1 flowing through the external load:

$$I_0 = I_1 + I_{R_0} \qquad \text{(Equation S1)}$$

By incorporating Ohm's Law, where V_{R0} denotes the internal voltage and R_0 represents the internal resistance, the equation can be transformed as follows:

$$I_0 = I_1 + \frac{V_{R_0}}{R_0} \qquad \text{(Equation S2)}$$

Given the external load resistance R_L, the equivalent reactance Z_R of its external circuit is:

$$Z_R = \sqrt{R_L^2 + \left(\frac{1}{2\pi f C_0}\right)^2} \qquad \text{(Equation S3)}$$

The internal voltage at this time is:

$$V_{R_0} = \frac{V_{R_L}}{R_L}\sqrt{R_L^2 + \left(\frac{1}{2\pi f C_0}\right)^2} \qquad \text{(Equation S4)}$$

Among them, V_{RL} is the voltage across the load resistor, f is the frequency of the AC signal passing through the internal capacitor, and the current I_0 of the equivalent current source model is:

$$I_0 = \frac{V_{R_L}}{R_L} + \frac{\frac{V_{R_L}}{R_L}\sqrt{R_L^2 + \left(\frac{1}{2\pi f C_0}\right)^2}}{R_0} \qquad \text{(Equation S5)}$$

Then, when a generator unit is in operation, the external load voltage $V_{RL,\,1U}$ can now be determined by (S6):

$$V_{\mathrm{RL,1U}} = \frac{I_0 R_0 R_L}{R_0 + \sqrt{R_{\mathrm{L}}^2 + \left(\frac{1}{2\pi f C_0}\right)^2}} \quad \text{(Equation S6)}$$

For four parallel units (4U), the equivalent parameters are modified as follows (parallel circuit rules): I_{4U}=4 I_{1U} , $R_{0,4U}$=$R_{0,1U}$/4, $C_{0,4U}$=4C_0. Substituting these into Equation (S6), the internal impedance of 4U is Equation (S7):

$$V_{\mathrm{RL,4U}} = \frac{I_0 R_0 R_L}{\frac{R_0}{4} + \sqrt{R_{\mathrm{L}}^2 + \left(\frac{1}{8\pi f C_0}\right)^2}} \quad \text{(Equation S7)}$$

According to Equation (S8), it can be seen that $V_{\mathrm{RL,4U}}$ is greater than $V_{\mathrm{RL,1U}}$, except for R_{L}, all denominators have decreased, but V_{RL} is actually the resistance of the electrostatic meter, approximately 200TΩ, much larger than the internal resistance R_0 of TENG. Therefore, there is a moderate increase in voltage.

$$V_{\mathrm{RL,4U}} = \frac{I_0 R_0 R_L}{\frac{R_0}{4} + \sqrt{R_{\mathrm{L}}^2 + \left(\frac{1}{8\pi f C_0}\right)^2}} > V_{\mathrm{RL,1U}} = \frac{I_0 R_0 R_L}{R_0 + \sqrt{R_{\mathrm{L}}^2 + \left(\frac{1}{2\pi f C_0}\right)^2}} \quad \text{(Equation S8)}$$

References

S1 Zhao, D.; Yu, X.; Wang, J.; Gao, Q.; Wang, Z.; Cheng, T.; Wang, Z. L. (2022). A Standard for Normalizing the Outputs of Triboelectric Nanogenerators in Various Modes. Energy Environ. Sci 15 (9), 3901–3911. https://doi.org/10.1039/d2ee01553f.

S2. Zhao, D., Yu, X., Wang, Z., Wang, J., Li, X., Wang, Z. L., & Cheng, T. (2021). Universal equivalent circuit model and verification of current source for triboelectric nanogenerator. Nano Energy, 89, 106335. https://doi.org/10.1016/j.nanoen.2021.106335

S3. P. Yin, L. Tang, Z. Li, H. Guo, and K. C. Aw, (2023) "Circuit representation, experiment and analysis of parallel-cell triboelectric nanogenerator," Energy Conversion and Management, vol. 278, p. 116741, Feb. 2023, doi: 10.1016/j.enconman.2023.116741.

S4. Zhao, D.; Li, H.; Wang, J.; Gao, Q.; Yu, Y.; Wen, J.; Wang, Z. L.; Cheng, T. A Drawstring Triboelectric Nanogenerator with Modular Electrodes for Harvesting Wave Energy. Nano Research 2023, 16 (8), 10931–10937. https://doi.org/10.1007/s12274-023-5796-6.
